# Genome‐wide adaptive evolution to underground stresses in subterranean mammals: Hypoxia adaption, immunity promotion, and sensory specialization

**DOI:** 10.1002/ece3.6462

**Published:** 2020-06-03

**Authors:** Mengwan Jiang, Luye Shi, Xiujuan Li, Qianqian Dong, Hong Sun, Yimeng Du, Yifeng Zhang, Tian Shao, Han Cheng, Weihua Chen, Zhenlong Wang

**Affiliations:** ^1^ School of Life Sciences Zhengzhou University Zhengzhou China; ^2^ College of Life Science and Technology Huazhong University of Science and Technology Wuhan China

**Keywords:** adaptation to hypoxia, adaptive evolution, immunity promotion, sensory specialization, subterranean mammals

## Abstract

Life underground has provided remarkable examples of adaptive evolution in subterranean mammals; however, genome‐wide adaptive evolution to underground stresses still needs further research. There are approximately 250 species of subterranean mammals across three suborders and six families. These species not only inhabit hypoxic and dark burrows but also exhibit evolved adaptation to hypoxia, cancer resistance, and specialized sensory systems, making them an excellent model of evolution. The adaptive evolution of subterranean mammals has attracted great attention and needs further study. In the present study, phylogenetic analysis of 5,853 single‐copy orthologous gene families of five subterranean mammals (*Nannospalax galili*, *Heterocephalus glaber*, *Fukomys damarensis*, *Condylura cristata*, and *Chrysochloris asiatica*) showed that they formed fou distinct clusters. This result is consistent with the traditional systematics of these species. Furthermore, comparison of the high‐quality genomes of these five subterranean mammalian species led to the identification of the genomic signatures of adaptive evolution. Our results show that the five subterranean mammalian did not share positively selected genes but had similar functional enrichment categories, including hypoxia tolerance, immunity promotion, and sensory specialization, which adapted to the environment of underground stresses. Moreover, variations in soil hardness, climate, and lifestyles have resulted in different molecular mechanisms of adaptation to the hypoxic environment and different degrees of visual degradation. These results provide insights into the genome‐wide adaptive evolution to underground stresses in subterranean mammals, with special focus on the characteristics of hypoxia adaption, immunity promotion, and sensory specialization response to the life underground.

## Graphical abstract

We download and evaluate the whole genome of subterranean mammals, and study the mechanisms of adaptative evolution to underground stresses of subterranean mammals through comparative genomics.

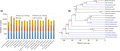

## 1 INTRODUCTION

Life underground has provided remarkable examples of adaptive evolution in subterranean mammals. There are approximately 250 species of subterranean mammals across three suborders, six families, 10 subfamilies, and 38 genera (Begall, Burda, & Schleich, 2007). Subterranean rodents inhabit burrows throughout their lives. Besides these rodents, some nonrodent species such as moles of the families Talpidae and Chrysochloridae are mainly distributed across Africa and Eurasia, also live underground (Begall et al., 2007). To adapt to the conditions of darkness and hypoxia in underground burrows, most subterranean mammals have evolved strategies and characteristics to cope with these environmental obstacles. For example, blind mole rats (*Spalax ehrenbergi*) can cope with extremely hypoxic environments of underground burrows during floods by overexpressing erythropoietin (EPO) and hypoxia‐inducible factor‐1α (HIF‐1α) in vivo (Imad, Aaron, & Eviatar, 2004). Furthermore, naked mole rats (*Heterocephalus glaber*) and blind mole rats show high cancer resistance (Gorbunova, Seluanov, Zhang, Gladyshev, & Vijg, 2014). In addition, some subterranean mammals exhibit specialized circadian rhythms (Ben‐Shlomo, Ritte, & Nevo, 1995), degenerative vision (Cooper, Herbin, & Nevo, 1993), improved hearing (Kim et al., 2011), and olfaction (Burda, Bruns, & Muller, 1990).

The development of high‐throughput sequencing technology has led to the use of comparative genomics methods by several studies to analyze the adaptive evolution to extreme environments, such as the adaptation of snub‐nosed monkeys to high altitudes (Yu et al., 2016) and the adaptive evolution of fish to extreme alkaline environments (Jian et al., 2016). Previous studies have used genomics to study the adaptive evolution of subterranean mammals, such as the adaptive complexes of blind mole rats to underground stresses (Fang, Nevo, et al., 2014; Fang, Seim, et al., 2014) and the adaptive evolution of vision‐ and skin‐related genes among subterranean mammalian species (Partha et al., 2017). In addition, comparison between subterranean mammals and closely related terrestrial rodents has revealed significant differences in the substitution rates of protein‐coding genes between both groups (Du, Yang, & He, 2015). The genomes and transcriptomes of subterranean mammals of three orders have shown that the number of positive selection genes shared among subterranean mammals was quite limited and that common signals of positive selection in sensory perception genes were not detected (Davies, Bennett, Faulkes, & Rossiter, 2018). However, further studies of genome‐wide adaptive evolution to underground stresses in subterranean mammals need to be conducted.

Therefore, in the present study, we performed comparative genomic studies on subterranean mammals and their closely related nonsubterranean species with high‐quality whole genome data using the hypothesis that subterranean mammals exhibit same or similarly functioning positively selected genes or expanded genes to deal with underground stresses.

## 2 MATERIALS AND METHODS

### 2.1 Data acquisition and processing of genomic data

Genomic sequences (FASTA format) and genome annotation information (GenBank format) of the 18 species assessed in the present study were retrieved from National Center for Biotechnology Information (NCBI) (https://www.ncbi.nlm.nih.gov) (Table S1).

The number of duplicated orthologous genes in most species was considerably high, making it difficult to identify their gene families. Therefore, we used the following method to filter variable shear among the 18 species. First, if a gene had multiple alternative splicing transcripts, only the longest transcript in the coding region was retained for subsequent analysis. Second, genes encoding proteins that are <30 amino acids long were excluded. We evaluated the completeness of the encoded gene sets using 4,104 Benchmarking Universal Single‐Copy Orthologs (BUSCO) groups of mammalian genes (Simão, Waterhouse, Ioannidis, Kriventseva, & Zdobnov, 2015).

### 2.2 Orthologous gene clustering

Orthologous genes from 18 species were automatically identified using OrthoMCL (version 5) (Fischer, Brunk, Chen, Gao & Stoeckert, 2011). The Smith–Waterman algorithm was used to compare all protein sequences in all‐against‐all comparisons and selected optimal alignment results with an e‐value cutoff of 1 × 10^−3^ for clustering. Moreover, the number of amino acids in each group of clustered protein sequences should be >30. Thereafter, the orthologous gene clusters were amplified using more similar genes within the same species, and copies with >97% sequence similarities were used. The longest transcript of each gene was obtained by searching the GenBank data of the NCBI gene sets.

### 2.3 Phylogenetic tree and divergence time estimation

Multiple alignments of the single‐copy orthologous genes from the 18 species were conducted using MUSCLE 3.8.31 (Edgar, 2004). Gblocks was used to remove ambiguously aligned blocks within the MUSCLE alignments (Talavera & Castresana, 2007). The alignments were finally combined to form a super alignment matrix. To select the best‐fitting amino acid substitution models, we applied ProtTest (Darriba etal., 2011) on the super alignment matrix. We established linked branch lengths for the analyses and used the corrected Akaike information criterion for final model selection. A phylogenetic tree of the 18 species was constructed using RAXML 8.2.4 (Stamatakis, 2014). Based on this phylogenetic tree, different subterranean mammals were grouped as follows: (a) each subterranean mammal forms a group with all other nonsubterranean mammals; (b) ≥2 subterranean mammals forming the same branch are grouped into a single group; (c) all nonsubterranean mammals together constitute a group. After grouping the different subterranean mammals, the phylogenetic trees of each group were also constructed using their own single‐copy orthologous genes for subsequent positive selection analysis.

The MCMCtree software from PAML was used to date the tree and calculate substitution rates (Yang, 2007). The age range of fossils was used to correct the timeline, which was obtained from the TimeTree website (http://www.timetree.org/). The operating parameters of MCMCtree were as follows: burn‐in = 10,000; sample number = 100,000; and sample frequency = 2.

### 2.4 Gene family analysis

The gene families shared among the compared species were selected to infer gene family expansion and contraction using CAFÉ (De Bie, Cristianini, Demuth, & Hahn, 2006). The global parameter *λ* was estimated using the maximum‐likelihood (ML) method based on the random birth and death model (Hahn, De Bie, Stajich, Nguyen, & Cristianini, 2005). *p* value was calculated by comparing each branch with its ancestor branch (Demuth, Bie, Stajich, Cristianini, & Hahn, 2006); a gene family with *p* < .05 indicated expansion.

### 2.5 Positive selection analysis

Positive selection analysis using the branch‐site model (models = 2; NS sites = 2; fix_blength = 2) was performed in all independent subterranean mammalian groups. Model A (fix_omega = 0) was compared with the null model (fix_omega = 1 and omega = 1), and the Codeml program in PAML was used to perform likelihood ratio tests. In each group, the branch of subterranean mammals (SMBs) and that of its most closely related species (control branch, CB) were used as foreground branches to detect the single‐copy orthologous alignment sequences of each group in their own phylogenetic tree with branch length (Table S2). Furthermore, the site model (models = 0; NS sites = 78) was used to conduct positive selection analysis of 13 nonsubterranean mammals (NSMs) to eliminate the influence of genes that are typically positively selected in mammals in the final results. After filtering out the positively selected genes (PSGs) of CB and NSMs, the final PSGs of SMBs were obtained. The *χ* ^2^ test with PAML was used to evaluate statistical significance. The fdrci function in the R software was used to correct for the false discovery rate in statistical computing.

### 2.6 Enrichment analysis of PSGs

ClusterProfiler (Yu, Wang, Han, & He, 2012) was used to perform GO and KEGG pathway enrichment analyses of the groups of PSGs of each subterranean mammal. The cutoff value was considered using single‐copy orthologous genes as the background with *p* < .01.

## 3 RESULTS AND DISCUSSION

### 3.1 Data processing and assessment

Using the NCBI (http://www.ncbi.nlm.nih.gov/gene) database, we downloaded the genomic data of 18 species (including five subterranean mammalian species belonging to the orders Rodentia, Afrosoricida, and Soricomorpha; nine aboveground rodent species belonging to the families Muridae, Caviidae, Octodontidae, Chinchillidae, Heteromyidae, Dipodidae, Sciuridae, and Cricetidae; and four species belonging to the orders Primates, Lagomorpha, and Carnivora). The GenBank accession numbers of these data are listed in Table S1. The duplicated homologous genes of all species were the longest transcripts of the respective gene. The result of the final BUSCO assessment of the filtered gene sets is presented in Table S1. The gene duplication rates for all species were <5%, and the complete‐ and single‐copy rates were >90%.

### 3.2 Phylogenetic tree

**FIGURE 1:**
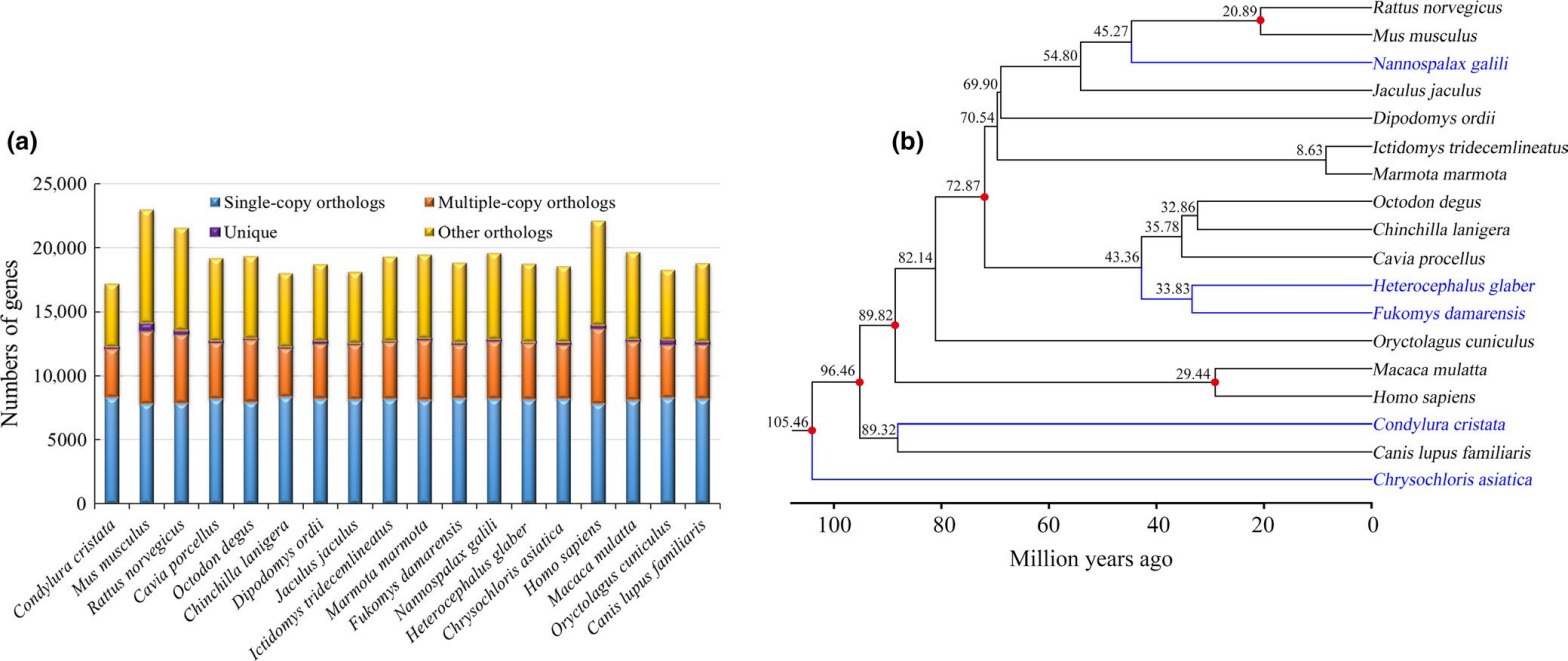
(a) Distribution of orthologous genes in different mammals. “Single‐copy orthologs” represents a single‐copy gene family; “Multiple‐copy orthologs” represents multiple‐copy gene families; “Unique orthologs” indicates genes specific to the corresponding species; and “Other orthologs” indicates genes other than those in the above categories. (b) Genome‐wide phylogenetic tree of 18 mammalian species. The number of nodes represents the estimated divergence time, and six red nodes are supported by fossil data. The bootstrap support rate for all nodes is 100

Overall, 19,172 orthologous gene families, including 5,853 single‐copy families, were identified from the 18 species (Figure 1a). Thereafter, an ML phylogenetic tree of the 18 species was constructed using the single‐copy orthologous genes. The phylogenetic tree indicated that the five subterranean mammals formed four distinct clusters (Figure 1b). This finding was consistent with the traditional systematics of these species (Bronner & Jenkins, 2005; Fang et al., 2015; Faulkes, Verheyen, Verheyen, Jarvis, & Bennett, 2010; Kock, Colleen, Laurence, Rodney, & Burda, 2006; Wilson & Reeder, 2005). In particular, *Fukomys damarensis* and *H. glaber* belonging to the Bathyergidae family formed a branch, whereas *Nannospalax galili*, *Condylura cristata*, and *Chrysochloris asiatica* each formed a branch supporting the families Spalacidae, Talpidae, and Chrysochloridae, respectively (Bronner et al., 2005; Fang et al., 2015; Faulkes et al., 2010; Kock et al., 2006; Musser, 2005). Among the five subterranean mammals, *C. asiatica* belonging to the Chrysochloridae family, which is distributed in southern Africa, was the first to diverge (105.46 million years ago); thereafter, *C. cristata* belonging to the Talpidae family, which is distributed in North America, diverged (89.32 million years ago). Then, *N. galili* diverged (33.83 million years ago) followed by *F. damarensis* and *H. glaber* (33.83 million years ago), with *N. galili* in southern Europe and *F. damarensis* and *H. glaber* in Africa (Figure 1b). These results suggest that in addition to forming four distinct clusters, the five subterranean mammals have a long evolutionary history spanning Africa, Asia, North America, and Europe, making them excellent models for the studying the adaptive evolution of subterranean mammals.

**FIGURE 2:**
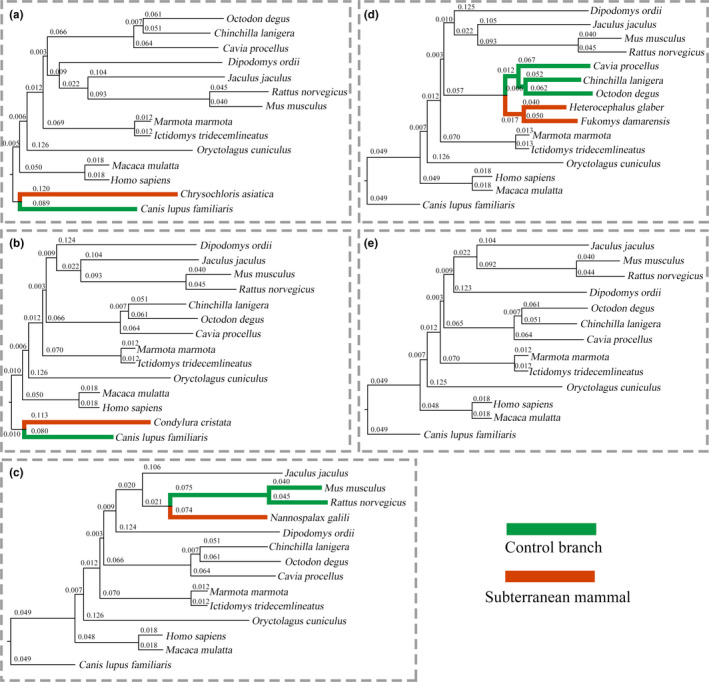
Phylogenetic tree with branch lengths of different groups of subterranean mammals. Phylogenetic tree of *Chrysochloris asiatica* and 13 nonsubterranean mammals. Phylogenetic tree of *Condylura cristata* and 13 nonsubterranean mammals. Phylogenetic tree of *Nannospalax galili* and 13 nonsubterranean mammals. Phylogenetic tree of *Fukomys damarensis* and *Heterocephalus glaber* and 13 nonsubterranean mammals. Phylogenetic tree of 13 nonsubterranean mammals

According to the phylogenetic location of the five subterranean mammals, the four groups of species for subsequent positive selection analysis were determined according to the methods described in Section 2.3. *F. damarensis* and *H. glaber*, belonging to the Bathyergidae family, were placed in one group, whereas the remaining three subterranean mammalian species were divided into different groups (Table S2). Accordingly, four different phylogenetic trees for each group were constructed using their own single‐copy orthologous genes with the branch length for subsequent positive selection analysis. Furthermore, the phylogenetic trees of 13 nonsubterranean mammals were reconstructed using the same method for identifying the genes that are commonly evolved in mammals to differentiate positive selection genes in subterranean mammals (Figure 2). The number of single‐copy orthologous genes in the groups “*C. asiatica* and the 13 NSMs (13NSM),” “*C. cristata* and the 13NSM,” “*N. galili* and the 13NSM,” “*F. damarensis*, *H. glaber*, and the 13NSM,” and “13NSM” was 6,497, 6,396, 6,536, 6,366, and 6,680, respectively (Table S3–S7).

### 3.3 Adaptive evolution of subterranean mammals

**FIGURE 3:**
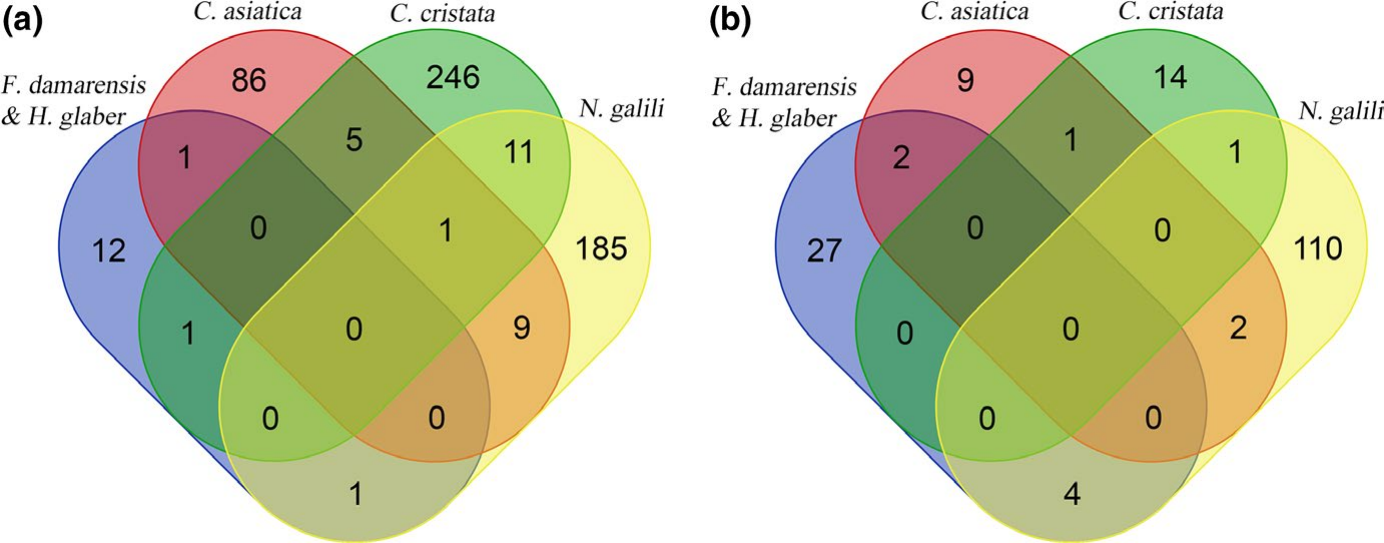
(a) Venn diagram of the number of positive selection genes in the four branches of subterranean mammals. (b) Venn diagram of the number of expanded gene families in the four branches of subterranean mammals

Positive selection and gene family expansion analyses were conducted to examine adaptive evolution. For positive selection analysis, the four groups of subterranean mammals and their respective control species were employed as foreground branches to identify the PSGs from their own single‐copy orthologous genes using the branch‐site model in PAML (Yang, 2007) (Tables S3–S6). Furthermore, the PSGs of the 13 nonsubterranean mammals were detected using only the site model to remove the PSGs of mammalian universal evolution (Tables S7). The results showed that there was no common gene among the four groups, and only two or three groups shared a few PSGs (Figure 3a).

For gene family expansion analysis, the 19,172 orthologous gene families of the branches of the 4 groups of subterranean mammals were examined and 14 (*C. asiatica*), 16 (*C. cristata*), 117 (*N. galili*), and 33 (*F. damarensis* and *H. glaber*) distinctly expanded gene families were detected (Tables S8–S11). No common expanded gene family was detected among the four groups of subterranean mammals (Figure 3b).

**FIGURE 4:**
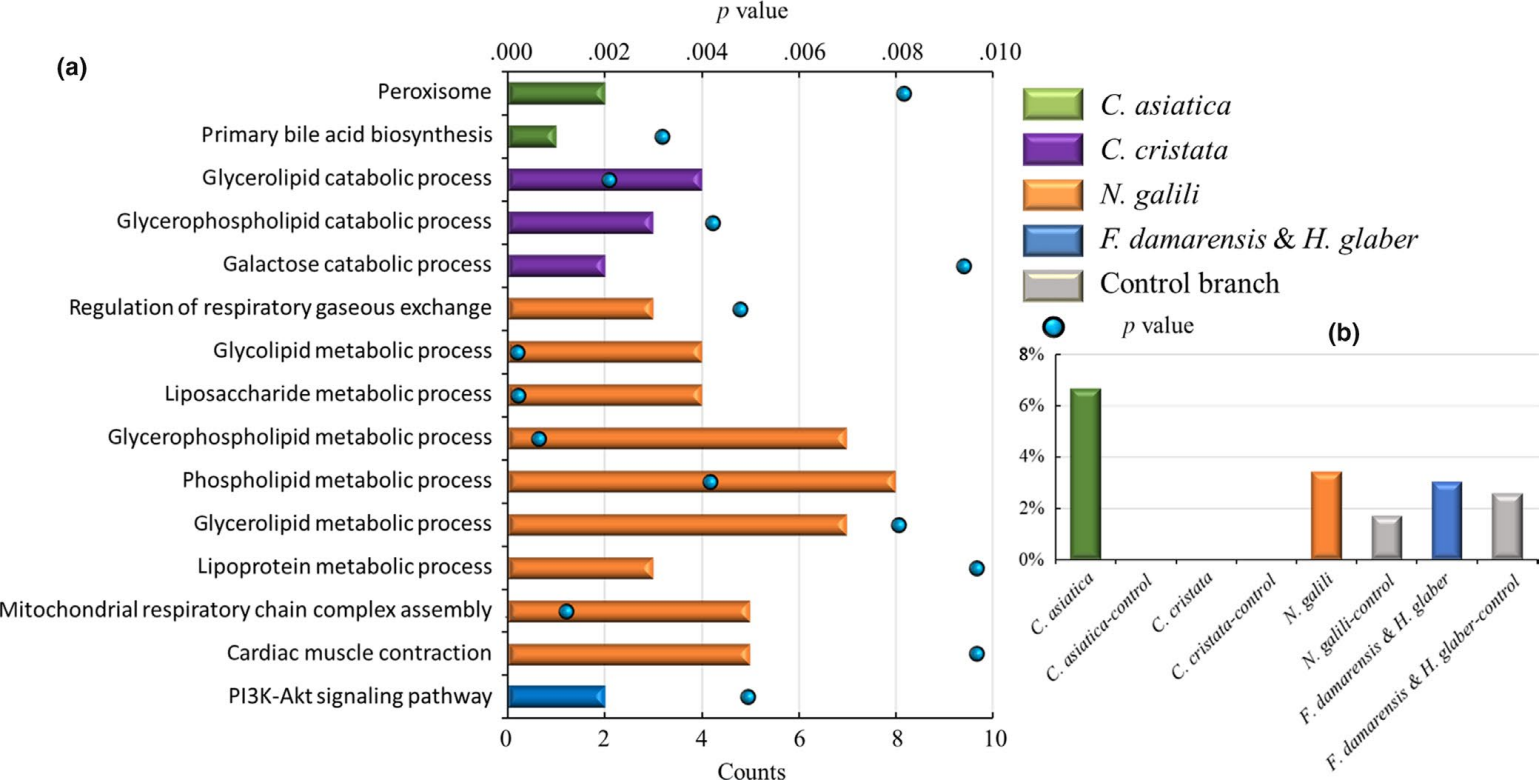
(a) Similar enrichment terms related to the positively selected genes for adaptation to hypoxia in the four branches of subterranean mammals. (b) Comparison of the percentages of expanded gene families related to adaptation to hypoxia in the four branches of subterranean mammals. Each color represents a subterranean branch. Notes: (a) Histogram represents the count of the terms, and the black dot represents the *p* value. (b) Histogram represents the percentage of expanded gene families

**FIGURE 5:**
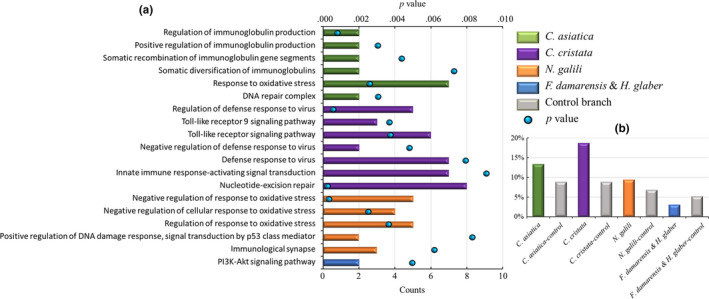
(a) Similar enrichment terms of the positively selected genes related to the immune system in the four branches of subterranean mammals. (b) Comparison of the percentages of expanded gene families related to the immune system in the four branches of subterranean mammals. Each color represents a subterranean branch. Notes: (a) Histogram represents the count of the terms, and the black dot represents the *p* value. (b) Histogram represents the percentage of expanded gene families

**FIGURE 6:**
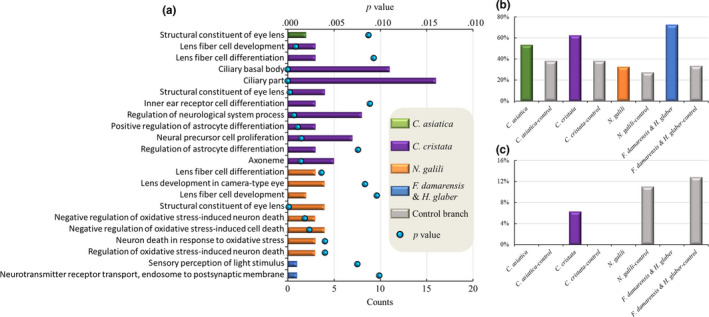
(a) Similar enrichment terms of the positively selected genes related to the sensory system in the four branches of subterranean mammals. (b) Comparison of the percentages of expanded gene families related to olfactory receptors in the four groups. (c) Comparison of the percentages of expanded gene families related to the vomeronasal receptor in the four groups. Each color represents a subterranean branch. Notes: (a) Histogram represents the count of the terms, and the black dot represents the *p* value. (b) and (c) Histograms represent the percentages of expanded gene families

Although common PSGs and expanded gene families among the four branches were rare, subsequent analysis indicated that the PSGs of each branch and the expanded gene families of each species showed enrichment in terms of several similar functional annotations that were directly related to the evolutionary trend of adaptation of these subterranean mammals to long‐term life in burrows (Table S12–S15, Figures 4, 5, 6).

#### 3.3.1 Hypoxia adaptation

Most mammals require oxygen to sustain, and hypoxic stress leads to a series of physiological issues. However, subterranean mammals can survive in burrows with a hypoxic environment for almost their entire life; this duration is longer than that observed for their related species. In our present study, the PSGs in all four branches of subterranean mammals exhibited enrichment of certain terms associated with hypoxia adaptation (Figure 4a). Regarding the *C. asiatica* branch, the enriched KEGG terms included peroxisome (map00120) and primary bile acid biosynthesis (map00120). Peroxisomes can be used for fatty acid oxidation, releasing a substantial amount of energy for facilitating body functions (Won, Park, Yun, Koh, & Lee, 2009). Bile acid is an important component of bile and plays an important role in fat metabolism (Hylemon et al., 2009) (Table S12a). Regarding the *C. cristata* branch, the enriched GO terms were glycerolipid catabolic process (GO:0046503), glycerophospholipid catabolic process (GO:0046475), galactose catabolic process (GO:0019388), and regulation of respiratory gaseous exchange (GO:0043576). The first three terms are related to glucolipid metabolism and play an important role in ensuring energy supply to the body (Timiras, 1977; Zhang et al., 2017) (Table S13a). For the *N. galili* branch, most of the enriched GO terms, such as mitochondrial respiratory chain complex assembly (GO:0033108) and liposaccharide metabolic process (GO:1903509), were related to energy supply. In addition, the GO term cardiac muscle contraction (GO:0060048) was significantly enriched in *N. galili*, which is related to cardiac protection in a hypoxic environment (Joyce et al., 2016) (Table S14a). For the *F. damarensis* and *H. glaber* branch, only one KEGG pathway—the PI3K/Akt signaling pathway (map04151)—was significantly enriched, which is closely related to the expressions of HIF‐1α and VEGF (Yang et al., 2009) (Table S15a).

Analysis of the expanded gene families revealed that the percentages of hypoxia‐related expanded gene families in the three branches of *C. asiatica*, *N. galili*, and *F. damarensis* and *H. glaber* were higher than those in their control branches (Figure 4b). Among these gene families, a common gene family that encodes the leucine‐rich repeat‐containing protein was detected between *C. asiatica* and *N. galili*; this protein aids in quicker glucose metabolism in the body, thereby providing energy to the tissues (Duan, Li, Liu, Li, & Yin, 2015). In addition, two other gene families were expanded in *N. galili*—one was the gene family encoding hemoglobin, which is a special type of protein involved in transportation of oxygen to the red blood cells (McCarthy, Vandegriff, & Winslow, 2001), whereas the other was the gene family encoding cytochrome C oxidase subunit 7C, which is closely related to respiratory electron transport, ATP synthesis by chemiosmotic coupling, and heat production by uncoupling proteins (Hüttemann, Schmidt, & Grossman, 2003). For the branch of *F. damarensis* and *H. glaber*, cytochrome P450 family 2 subfamily J was expanded, which has been shown to have a diverse range of effects on the vasculature, including regulation of inflammation, vascular tone, cellular proliferation, angiogenesis, and metabolism (Askari, Thomson, Edin, Zeldin, & Bishop‐Bailey, 2013).

Our study suggests that maintaining the body's energy supply under hypoxic environments is an adaptive trait shared by *C. asiatica*, *C. cristata*, and *N. galili*. In addition, the four branches of subterranean mammals exhibit their own mechanisms of hypoxia adaptation. For example, *C. cristata* shows hypoxia adaptation by regulating respiratory gas exchange, whereas *N. galili* shows this adaptation by increasing myocardial contraction and hemoglobin transport capacity. For *F. damarensis* and *H. glaber*, this adaptation occurs mainly via the regulation of the hypoxia‐inducible factor pathway and gene expansion related to angiogenesis. Differences in species distribution and climate lead to variations in oxygen levels in burrows, thereby resulting in different adaptive mechanisms. For instance, naked mole rats adapt to hypoxic environments by actively reducing their body temperature and activity (Kim et al., 2011). On the other hand, blind mole rats adapt to severe hypoxia via mutations in *VGF* and *EPO* as well as alterations in the microvascular system (Fang et al., 2015). *Lasiopodomys mandarinus* adapts to hypoxia by enhancing its oxygen transport capacity and modulating oxygen consumption (Dong et al., 2018). Due to differences in soil hardness and environmental climate, the burrow systems of subterranean mammals are not always completely enclosed. In addition, unlike *N. galili*, not all subterranean mammals refrain from never climbing out of the burrow, some occasionally climb out for foraging; therefore, hypoxic stress faced by these species is different. Subterranean mammals, such as *N. galili*, spend their entire lives in an oxygen‐deficient burrow system; therefore, more sophisticated evolutionary approaches may require multiple methods to adapt to the hypoxic environment. However, in subterranean mammals inhabiting burrow systems with lower oxygen levels or in those that climb out occasionally, the evolution of angiogenesis possibly already helps in coping with hypoxic environments.

#### 3.3.2 Immunity promotion

Several studies have described cancer resistance in some subterranean mammals (Azpurua & Seluanov, 2013; Gorbunova et al., 2012, 2014), suggesting that this ability is common in these species. The naked mole rat has a maximum lifespan of 30 years and a low incidence of neoplasia (Buffenstein, 2008; Edrey, Martha, Mario, James, & Rochelle, 2011). Similarly, the blind mole rat exhibits longevity and cancer resistance (de Magalhães & Costa, 2010; Nasser et al., 2009). In the present study, for *C. asiatica*, the enriched GO terms were DNA repair complex (GO:1990391), which can allow tumor cells to survive chemotherapy‐induced DNA damage (Helleday, Petermann, Lundin, Hodgson, & Sharma, 2008), response to oxidative stress (GO:0006979), which is closely associated with chronic inflammation and cancer (Reuter, Gupta, Chaturvedi, & Aggarwal, 2010), and terms associated with immunoglobulin‐related mechanisms, including regulation of immunoglobulin production (GO:0002637), positive regulation of immunoglobulin production (GO:0002639), somatic recombination of immunoglobulin gene segments (GO:0016447), and somatic diversification of immunoglobulins (GO:0016445) (Table S12a). For *C. cristata*, the enriched GO terms were nucleotide excision repair (GO:0006289), which specifically protects against mutations indirectly caused by environmental carcinogens (Friedberg, 2001), as well as terms associated with toll‐like receptor signaling, including the toll‐like receptor signaling pathway (GO:0002224) and toll‐like receptor 9 signaling pathway (GO:0034162); which are known to have crucial roles in the realization of innate and adaptive immune response; in cell proliferation, survival, apoptosis, angiogenesis, and tissue remodeling and repair (Kutikhin, 2011); in defensive responses to viruses, including regulation of the defense response to viruses (GO:0050688), negative regulation of the defense response to viruses (GO:0050687), and defense response to viruses (GO:0051607); and in innate immune response‐activating signal transduction (GO:0002758). However, Epstein–Barr virus infection (map05169) was found in the KEGG enrichment results when control species were used as the foreground branch (Table S13a). For *N. galili*, the enriched GO terms were positive regulation of DNA damage response; signal transduction by p53 class mediator (GO:0043517), of which the tumor suppressor p53 mainly induces cell cycle arrest/DNA repair or apoptosis in the DNA damage response (Zhang, Liu, & Wang, 2011); immunological synapse (GO:0001772), which has been termed owing to its functional analogy to the site of intercellular information transfer between neurons (Delon & Germain, 2000); as well as three negative regulation of response to oxidative stress terms including negative regulation of response to oxidative stress (GO:1902883), negative regulation of cellular response to oxidative stress (GO:1900408), and regulation of response to oxidative stress (GO:1902882) (Table S14a). With regard to the *F. damarensis* and *H. glaber* branch, the enriched KEGG pathways were mainly in the PI3K/Akt signaling pathway (map04151), which is one of the most important signal transduction pathways in cells and plays a key role in promoting cell proliferation and inhibiting apoptosis by affecting the activation status of multiple downstream effector molecules. This pathway is closely involved in the development of various human cancer types (Table S15a). When control species were used as the foreground branch, the positive regulation of interleukin‐2 biosynthetic process (GO:0045086) was enriched (Table S15b).

In the expanded gene family, we found that eight branches of all the four groups of subterranean mammalian species exhibited immune‐related gene families, indicating that immune‐related gene family expansion is ubiquitous in mammals. However, except for the *F. damarensis* and *H. glaber* group, the subterranean mammalian branches contained a higher percentage of the expanded immune‐related gene families than the control group (Figure 5b). The expanded gene family in the *F. damarensis* and *H. glaber* branch was an immunoglobulin gene family, whereas that in the control branch was the tripartite motif and CD33 gene families. The tripartite motif gene family was also expanded in *C. cristata*. Recent studies have shown that the tripartite motif gene family is involved in the regulation of innate immune responses via the modulation of pattern recognition receptor signaling pathways (Kawai & Akira, 2011). In addition, the MHC class I family is expanded in both the *C. asiatica* and *N. galili* branches. MHC class I molecules regulate responsiveness and receptor repertoire formation in natural killer cells (Höglund & Brodin, 2010). For *N. galili*, the B7 gene family, comprising activating and inhibitory co‐stimulatory molecules that positively and negatively regulate immune responses, was expanded (Zou & Chen, 2008).

All the four groups of subterranean mammalian species exhibited positive selection or expansion of immune‐related genes. Furthermore, oxidative stress was significantly enriched in *C. asiatica*, *C. cristata*, and *N. galili*. DNA repair and T‐cell‐related genes were detected in both *C. asiatica* and *C. cristata*. In addition to a common anticancer approach, subterranean mammals in each branch exhibited their own special approaches. The enrichment results of the *C. cristata* branch included toll signals and multiple virus defense terms, whereas those of the control species included viral infection. For the *N. galili* branch, the main tumor suppressor gene p53 facilitated DNA damage regulation. The mechanisms of adaptation in the *F. damarensis* and *H. glaber* branch were mainly regulated by the PI3K/Akt signaling pathway and immunoglobulin expansion, whereas those of its control branch had only one enriched term about interleukin‐2‐related immunity. These results suggest that cancer resistance is a universal phenomenon among subterranean mammals; however, the anticancer patterns and types of each subterranean mammal are quite different. Typically, subterranean mammals exhibit high cancer resistance in different organs or tissues via variable mechanisms.

#### 3.3.3 Sensory specialization

Subterranean mammals have a special sensory system, degraded vision, special hearing function, and developed olfactory sense. Most of them have small eyes and poor vision, and some of them are blind (blind mole rat) (Herbin, Repérant, & Cooper, 1994; Herbin et al., 1995; Heth & Todrank, 2000; Kott, Sumbera, & Nemec, 2010; Němec et al., 2008). Moreover, although visual perception is maintained, they show different degrees of visual degradation (Herbin et al., 1994). To communicate with each other, subterranean mammals bang their heads on the walls of the burrows (Rado et al., 1987). Furthermore, they can only hear sounds of a certain frequency and wavelength (Bronchti, Heil, Scheich, & Wollberg, 1989; Schleich, Veitl, Knotková, & Begall, 2007). Subterranean mammals primarily feed on underground plant roots and worms, and a keen sense of smell improves their ability to find food underground (Heth & Todrank, 2007).

The PSGs in each subterranean mammal branch were enriched in terms of the structural constituent of the visual or auditory system and the regulation and development of the sensory nervous system (Figure 6a). With regard to *C. asiatica*, the enriched GO terms were structural constituents of the eye lens (GO:0005212) (Table S12a). For *C. cristata*, there were five enriched terms related to the visual system and one enriched term related to the auditory system: lens fiber cell development (GO:0070307), lens fiber cell differentiation (GO:0070306), ciliary basal body (GO:0036064), ciliary parts (GO:0044441), structural constituents of the eye lens (GO:0005212), and inner ear receptor cell differentiation (GO:0060113). Furthermore, there were five significantly enriched terms related to the nervous system for *C. cristata*: regulation of neurological system processes (GO:0031644), positive regulation of astrocyte differentiation (GO:0048711), neural precursor cell proliferation (GO:0061351), regulation of astrocyte differentiation (GO:0,048,710), and regulation of axoneme differentiation (GO:0005930). Of these, astrocytes are increasingly considered to play an important role in neurovascular coupling (Mccaslin, Chen, Radosevich, Cauli, & Hillman, 2011) (Table S13a). With regard to *N. galili*, there were four enriched terms related to the visual system: lens fiber cell differentiation (GO:0070306), lens development in the camera‐type eye (GO:0002088), lens fiber cell development (GO:0070307), and structural constituents of the eye lens (GO:0005212). Furthermore, there were four enriched terms related to the nervous system: negative regulation of oxidative stress‐induced neuron death (GO:1903204), negative regulation of oxidative stress‐induced cell death (GO:1903202), neuron death in response to oxidative stress (GO:0036475), and regulation of oxidative stress‐induced neuron death (GO:1903203) (Table S14a). With regard to *F. damarensis* and *H. glaber*, the main enriched term related to the visual system was sensory perception of light stimuli (GO:0050953) and that related to the nervous system was neurotransmitter receptor transport, endosome to postsynaptic membrane (GO:0098887) (Table S15a).

In all the four groups, several olfactory receptor gene families were expanded, and the percentages of the expanded immune‐related gene families in the four branches of subterranean mammalian species were all higher than those of the control branches (Figure 6b). In the four groups of subterranean mammals, 53.33% (8), 62.50% (10), 32.48% (38), and 72.73% (24) of olfactory receptor gene families were expanded in the *C. asiatica*, *C. cristata*, *N. galili*, and *F. damarensis* and *H. glaber* branches, respectively. On the other hand, the vomeronasal receptor gene families were expanded only in the *C. cristata* and in *F. damarensis* and *H. glaber* branches.

Regarding the gene expansion of olfactory receptors, all the four branches of subterranean mammalian species showed changes in eye structure or optical signal transmission systems. Similar to hypoxia adaptation, due to the different lifestyles of different subterranean mammals, some subterranean mammals live in burrows for their entire lifetime, and they are completely blind. Furthermore, some subterranean mammals occasionally climb out of their burrows for foraging and have varying degrees of visual degradation. Completely blind subterranean mammals cannot receive light signals, whereas subterranean mammals with visual degradation can receive light signals and only have dysfunctions associated with the neurotransmission system. Moreover, our findings indicate that the formation of visual degradation and special auditory system of subterranean mammals occurs during their development. These results are consistent with those reported by Sanyal, Jansen, Grip, Nevo, and Jong (1990) that the visual structures of blind mole rats are normal in early embryos, whereas photoreceptors and neurons are structurally degraded in adults. An acute sense of smell improves the ability to find food underground (Heth & Todrank, 2007), as observed in Zambian mole rats (*Cryptomys anselli* and *C. kafuensis*) (Lange, Neumann, Hagemeyer, & Burda, 2005), members of the Bathyergidae family (Patzenhauerová, Bryja, & Šumbera, 2010), and blind mole rats (Fang, Nevo, et al., 2014; Fang, Seim, et al., 2014).

Rodents rely on specialized visual, auditory, and olfactory systems for communication, foraging, navigation, and predator avoidance (Hindley, Nelson, Aggleton, & Vann, 2014; Youngstrom & Strowbridge, 2012; Zolotykh & Kozhevnikova, 2017). However, following the adaptation of subterranean mammals to long‐term underground life, their sensory systems have been modified (degraded vision as well as specialized auditory and olfactory systems) perhaps during the adaptive evolution of these species.

## 4 CONCLUSIONS

Comparative analysis of high‐quality genomes shows that the PSGs are species specific and are not shared among the five subterranean mammals. The PSGs all have similar functional enrichment categories, including hypoxia tolerance, immunity promotion, and sensory specialization, which adapted to the environment of underground stresses. These results indicate that the subterranean mammals may adapt to life underground through the convergent functional category of the PSGs rather than the PSGs themselves. It is noteworthy that for hypoxia tolerance and visual degradation, different subterranean mammals have individual lifestyles and experience different soil hardness and climate, resulting in different frequency and amount of hypoxic stress and light source stimulation; therefore, different subterranean mammals have evolved different pathways to adapt to their hypoxic environment as well as to the different degrees of visual degradation. Our results confirm that adaptive evolution of subterranean mammals at the genome‐wide level evolves similar functions via different molecular evolution, possibly reflecting the important role of adaptive evolution to subterranean stresses and providing help for further studies on the adaptive evolution of subterranean mammals.

## CONFLICT OF INTEREST

The authors declare no competing interests.

## AUTHOR CONTRIBUTION

**Zhenlong Wang:** Conceptualization (equal); funding acquisition (equal); resources (equal); supervision (equal). **Mengwan Jiang:** Data curation (equal); formal analysis (equal); funding acquisition (equal); methodology (equal); project administration (equal); software (equal); supervision (equal); validation (equal); visualization (equal); writing – original draft (equal); writing – review & editing (equal). **Luye Shi:** Validation (equal); visualization (equal); writing – original draft (equal); writing – review & editing (equal). **Xiujuan Li:** Formal analysis (equal); investigation (equal); software (equal). **Qianqian Dong:** Formal analysis (equal); investigation (equal). **Hong Sun:** Formal analysis (equal); investigation (equal). **Yimeng Du:** Investigation (equal). **Yifeng Zhang:** Investigation (equal); visualization (equal). **Tian Shao:** Funding acquisition (equal); investigation (equal). **Han Cheng:** Formal analysis (equal); funding acquisition (equal); methodology (equal); software (equal). **Weihua Chen:** Formal analysis (equal); investigation (equal); methodology (equal); software (equal); validation (equal).

## Data Availability

The supplemental tables available at FigShare (https://doi.org/10.6084/m9.figshare.10310492.v3). Table S1 shows the BUSCO and GenBank accession numbers of the genomes of the 18 species. Table S2 shows the groups of the PAML test. Tables S3–S7 contain the lists of positively selected genes of all PAML tests. Tables S8–S11 contain the expanded gene families of the four subterranean mammal's branches. Tables S12–S15 contain the GO‐ and KEGG‐enriched terms of the positively selected genes of all PAML tests.
